# Effect of temperature on the hydrolysis of levan treated with compressed hot water fluids

**DOI:** 10.1002/fsn3.1488

**Published:** 2020-03-14

**Authors:** Naoto Shimizu, Andres Abea, Tetsuya Ushiyama, Ebru Toksoy Öner

**Affiliations:** ^1^ Research Faculty of Agriculture Hokkaido University Sapporo Japan; ^2^ Field Science Center for Northern Biosphere Hokkaido University Sapporo Japan; ^3^ Graduate School of Agriculture Hokkaido University Sapporo Japan; ^4^ Department of Bioengineering IBSB Marmara University Istanbul Turkey

**Keywords:** compressed hydrolysate, fructooligosaccharides, levan, light scattering

## Abstract

The hydrolysis of levan using compressed hot water for the production of functional fructooligosaccharides (FOSs) was investigated. Levans from *Erwinia herbicola* (EH) and *Halomonas smyrnensis* (HS) were characterized using scanning electron microscopy and light scattering techniques, and hydrolyzed using compressed hot water at four temperatures (120, 140, 160, and 180°C). The hydrolysates were analyzed using high‐performance liquid chromatography and electrospray ionization‐mass spectrometry. Levan HS showed a crystalline morphology, whereas levan EH showed an aggregated structure. Both levans had molar masses on the order of 10^6^ g/mol, but levan EH had a smaller radius of gyration, hydrodynamic radius, and intrinsic viscosity. Levan EH hydrolyzed into FOSs at approximately 120°C, whereas levan HS required a temperature of at least 160°C, possibly because of differences in the degree of branching of the two levans. Both samples were degraded to fructose when treated at 180°C.

## Graphical abstract

Levans from *Erwinia herbicola* (EH) and *Halomonas smyrnensis* (HS) were characterized using scanning electron microscopy and light scattering techniques and then hydrolyzed using compressed hot water.

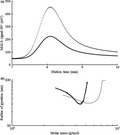

## 1 INTRODUCTION

Developing sustainable processes and designing new chemical entities that improve the efficiency and reduce the environmental impact of chemical transformations are important. Carbohydrates are the most abundant class of natural compounds, displaying a diverse range of structures and affording multiple possible transformations into industrial products (Rauter, Vogel, & Queneau, 2010).

Levan is a fructose‐based homopolysaccharide, which is a fructan composed primarily of β‐D‐fructofuranose residues linked by β‐(2‐6) glycosidic bonds with occasional β‐(2→1) branching and terminal glucose units. Plants and microbes produced levan‐type fructans. Short‐chain levans are synthesized in the vacuoles of plants, whereas long‐chain levans are produced by microorganisms as extracellular polysaccharides (Toksoy Öner, Hernández, & Combie, 2016).

Besides their role as important storage carbohydrates in higher plants, fructans have been recognized recently as multifunctional compounds involved in biotic and abiotic stress resistance and signaling (Versluys, Kirtel, Toksoy Öner, & Ende, 2018). Microbial levan can serve a variety of purposes, including forming an important component of biofilms, shielding microorganisms from desiccation, increasing the virulence of plant pathogens, forming an oxygen diffusional barrier, and serving as an extracellular nutrient reservoir. Unlike other natural polymers, levan self‐assembles into spherical colloids with low intrinsic viscosity and high biocompatibility (Adnan Erkorkmaz, Kirtel, Duru, & Toksoy Öner, 2018). “Green” processes involving the conversion of microbial levan into industrial products and (particularly) food additives offer further possibilities for sustainable commercial development (Ortiz & García, 2010).

Blake, Clarke, Jansson, and McNeil (1982) investigated levan production from a strain of the gram‐negative bacterium *Erwinia herbicola* (*E. herbicola*) (isolated from a crushing mill at a sugar factory), and this product is now commercially available. *Halomonas smyrnensis* (*H. smyrnensis*) AAD6T was reported by Poli et al. (2009) as the first halophilic, levan‐producing microorganism, facilitating levan production under non‐sterile conditions by use of high‐salinity medium (Adnan Erkorkmaz et al., 2018).

Obtaining chemicals from biomass commonly requires, as a first step, hydrolysis of the biomass to fundamental compounds (Vaquerizo, Abad, Mato, & Cocero, 2018). Fructooligosaccharides (FOSs) obtained from the hydrolysis of fructans can be used as additives in the food industry, for example, as sweeteners or dietary fiber. FOS can be considered a functional food ingredient given its capacity to improve gut absorption of calcium and magnesium, prevent urinal infections (by promoting proliferation of lactobacilli), reduce the risk of colon cancer, enhance lipid metabolism, and attenuate the development of tooth decay (Patel & Goyal, 2011).

Commonly employed methods of extraction of oligosaccharides are hydrolysis of fructans or synthesis from disaccharide substrates by enzymatic and chemical treatment (Patel & Goyal, 2011). FOSs are produced industrially from sucrose‐based substrates by microbial enzymes possessing transfructosylating activity; however, given the low yields of such biotransformations, commercially available FOSs may contain sucrose, fructose, and glucose at concentrations exceeding 500 g/kg of total FOS dry weight (Sánchez, Guio, Garcia, Silva, & Caicedo, 2008).

Treatment with compressed hot water is an alternative process for more sustainable hydrolysis of polysaccharides than processes that use acids or enzymes. Traditional hydrolytic methods have some disadvantages, including low selectivity, long reaction times, and the generation of residual effluents. The use of compressed hot water decreases both the reaction times and the equipment volumes, permitting water to be the only residual effluent (Vaquerizo et al., 2018). Near the critical point, water is an excellent solvent for organic compounds and its ionization constant (*K*_w_) is approximately three orders of magnitude higher than that of ambient liquid water, which makes heated water a good medium for both acid‐ and base‐catalyzed reactions of organic compounds (Savage, 1999).

A major challenge in obtaining valuable compounds from biomass is controlling the extent of the hydrolysis reaction. Thus, a deep understanding of the hydrolysis mechanisms and fine control of the reaction conditions are necessary (Vaquerizo et al., 2018). Previous work has examined the use of compressed hot water in the hydrolysis of fructans like inulin (Itoh & Shimizu, 2014; Shimizu, Ushiyama, & Itoh, 2019); however, the use of this reagent in the hydrolysis of levan has not (to our knowledge) been documented previously. In the present study, compressed hot water fluids were applied to levans from two different bacterial sources. The reaction products obtained from each substrate at different process temperatures were characterized to assess the feasibility of this method for FOS production.

## 2 MATERIALS AND METHODS

### 2.1 Materials

Two bacterial levans from *E. herbicola* (Sigma‐Aldrich Co.) and from *H. smyrnensis* were used as the hydrolysis substrates. Levan from *H. smyrnensis* was provided by the Industrial Biotechnology and Systems Biology research group at the Department of Bioengineering of Marmara University. Hereafter, levan from *E. herbicola* and *H. smyrnensis* are referred to as levan EH and levan HS, respectively.

### 2.2 Levan hydrolysis with compressed hot water fluids

**Figure 1:**
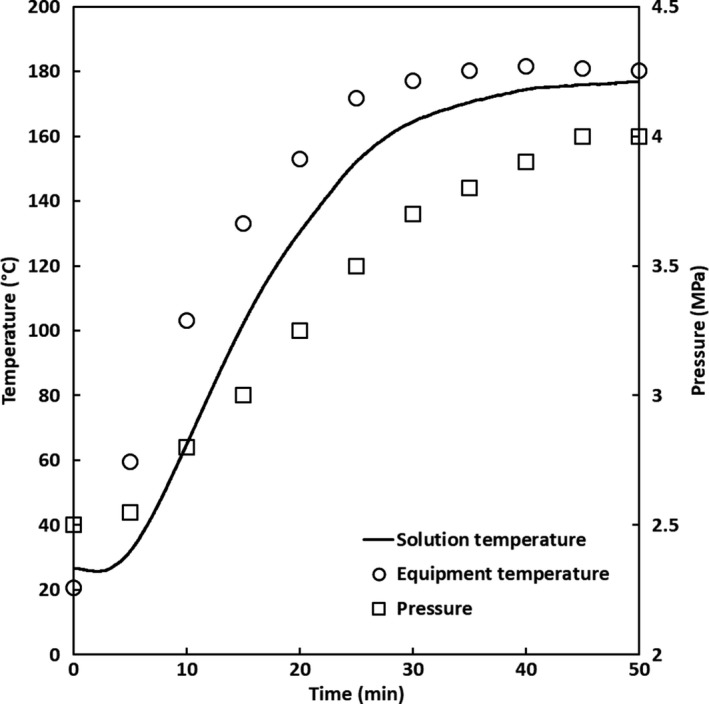
Typical behavior of the equipment temperature, solution temperature, and pressure during the hydrolysis of levan using compressed hot water fluids

Levan hydrolysis was performed as reported previously by Shimizu et al. (2019) and Yoshioka and Shimizu (2014) in a batch‐type reactor equipped with a pressure‐resistant vessel made from SUS‐316 stainless steel and an inner container made from Teflon equipped with a type‐K thermocouple and a pressure gauge. Levan (0.02 g), ultrapure water (10 ml), and a magnetic stir bar were placed into the inner container, and the vessel was pressurized to the appropriate pressure with nitrogen gas. The concentration was chosen by considering the solubility of the sample to avoid conglomerations and the sensitivity of the HPLC system detectors. The vessel was sealed and then stirred at 17 *g* while heating in an organic synthesizer (Chemi Station PPV‐3000) to temperatures of 120, 140, 160, or 180°C. Upon achieving the desired temperature, the vessel was maintained under those conditions for 15 min. The temperature inside the vessel was monitored at 1‐s intervals by using the thermocouple. The temperature of the heating block and the pressure inside the vessel were monitored every 5 min; the typical behavior of these parameters during the hydrolysis is shown in Figure 1. The reaction was quenched by rapidly cooling the vessel with chilled water (5°C) when the preparation was complete. The resulting levan hydrolysate solutions were collected in glass vials and stored at 5°C pending analysis.

### 2.3 Characterization of levan raw materials

#### 2.3.1 Scanning electron microscopy (SEM)

Both levan samples were observed using SEM (JSM‐6301F, JEOL Ltd.). The samples were mounted onto aluminum stubs with double‐sided carbon tape and coated with gold‐palladium alloy using an ion‐sputtering apparatus (E101 ION SPUTTER, Hitachi, Ltd.) before SEM observation. Observations were carried out under high‐vacuum conditions (below 1.0 × 10^–3^ Pa) at an acceleration voltage of 10 kV and a magnification ratio of 60–150×.

#### 2.3.2 Multi‐angle light scattering (MALS) analysis

Size‐exclusion chromatography (SEC)‐MALS was composed of a high‐performance liquid chromatography (HPLC) instrument (HPLC 1200 Infinity series; Agilent Technologies), a MALS detector (MALS DAWN8+; Wyatt Technology), and a differential refractive index (RI) detector (OptilabrEX, Wyatt Technology). The MALS and RI used a wavelength of 658 nm. The HPLC was equipped with a liquid‐delivery pump, an automatic sampler, a column compartment, and a UV detector (280 nm). A LB‐G6B guard column and a LB‐806M analytical size‐exclusion column (Showa Denko K.K.) were connected in tandem. The columns were held at 30°C within the column compartment. The buffer solution for separation was 50 mol/L NaNO_3_ in ultrapure water that was passed through a 0.45‐µm filter (MF‐Millipore membrane filters; Merck Millipore Co., Carrigtwohill Co.) before use. A 100‐µl sample was injected at a flow rate of 1.0 ml/min after the laser intensity for detection in MALS and RI was stable. The pullulan standard solution (PSS‐dpul50k; PSS polymer Standard Service GmbH) for detector signal alignment was used at a concentration of 3 g/L.

Processing of the SEC‐MALS data was performed using ASTRA software 5.3.4 (Wyatt Technology). The collection interval in MALS was 0.5 s. A RI increment (dn/dc) of 0.138 was used for levan, as reported by Sennaroglu et al. (2014). The molar mass and the radius of gyration (*R*_g_) were determined by Zimm plots (Zimm, 1948). The data from the MALS detector were fitted to a straight line in the Zimm plots. Inaccurate data, especially those obtained from the low‐scattering‐angle detector, were not used. The second coefficient was set to zero because of the low concentration of the samples. ASTRA 5.3.4 was used for following calculations by integration over one peak:

(1) $$\text{Number average molar mass}\;M_{n}=\sum c_{i}/\sum \left(c_{i}/M_{i}\right)$$

(2) $$\text{Weight average molar mass}\;M_{w}=\sum \left(c_{i}M_{i}\right)/\sum c_{i}$$

(3) $$z\;\text{average mean square radius}\;Rg^{2}=\sum \left(c_{i}M_{i}{〈r^{2}〉}_{i}\right)/\sum \left(c_{i}M_{i}\right)$$

where *c_i_*, *M_i_*, and ${〈r^{2}〉}_{i}$ are the mass concentration, molar mass, and mean square radius of the *i*th slice, respectively.

The polydispersity index was calculated as follows:

(4) $$\text{PD}=\frac{M_{w}}{M_{n}}$$

The molecular shape information was obtained from the slope of the conformation plot, which plots the logarithm of the *R*_g_ as a function of the logarithm of the *M_i_*:

(5) $$R_{g}=kM_{i}^{v}$$

The value of the exponent *v* can be obtained from the slope of log *R*_g_ versus log *M_i_*. The molecular structure can then be estimated from this slope. For a spherical structure, *v* = 0.33; for a rod structure, *v* = 1; and for a random coil structure, *v* = 0.5–0.6 (Shimizu & Ushiyama, 2018).

#### 2.3.3 Dynamic light scattering (DLS) analysis

The hydrodynamic radius was obtained with a fiber DLS instrument (FDLS‐3000; Otsuka Electronics Co., Ltd.). The sample solutions were placed in 12‐mm‐diameter glass cells (Otsuka Electronics Co. Ltd.) that had been cleaned with chloroform. The measurement device was filled with silicone and held at 25°C using a thermostat. A 532‐nm laser was used, and the measurement angle was 90°. The hydrodynamic radius (*R*_h_) was obtained using the Stokes–Einstein equation:

(6) $$R_{h}=\frac{\mathrm{kT}}{6\pi \eta D}$$

where *k* is Boltzmann's constant, *η* is the viscosity of the solvent, *D* is the diffusion coefficient, and *T* is the absolute temperature. Three hundred particles were counted for each substrate. The *R*_h_ distribution and the volume average particle size were obtained using the equipment's CONTIN routine.

#### 2.3.4 Levan solution preparation for sample characterization

Bacterial levans and ultrapure water were mixed by a magnetic stirrer using an organic synthesizer (Chemi station PPV 3000, Tokyo Rikakikai Co., Ltd.) with an agitation speed of 500 rpm at 80°C for 30 min. The concentration was 0.1% (w/v). Sodium azide (0.02%) was added to the solutions to avoid bacterial spoilage. The mixtures were stored overnight at 5°C, and then, two samples of each levan source were filtered (0.45‐µm pore size; Millipore Co.) and maintained at 5°C pending dynamic light scattering (DLS) and multi‐angle light scattering (MALS) measurements. Mean values of various other parameters for both levan sources were calculated as described below. All the experiments were performed in duplicate.

#### 2.3.5 Intrinsic viscosity

Viscosity of levan solutions ranging from 0.002 to 0.01 g/ml was measured at 25°C using an Ostwald type capillary viscometer (026300‐1; Sibata Scientific Technology, Ltd.) suspended in a thermostatic water bath under precise temperature control. Using exactly 8 ml of sample, three efflux time readings were obtained at each concentration. Values of relative viscosity (*η*_esp_) and specific viscosity (*η*_rel_) were obtained as follows:

(7) $$\eta_{\text{rel}}=\frac{t}{t_{s}}$$

(8) $$\eta_{\text{esp}}=\frac{t-t_{s}}{t_{s}}=\eta_{\text{rel}}-1$$

where *t* is the efflux time of each solution, and *t*_s_ is the efflux time of the solvent (ultrapure water). Concentrations were chosen to obtain a specific viscosity around 0.1 for the most dilute solution (Podzimeck, 2011) to assure good accuracy and linearity. The intrinsic viscosity was calculated as follows.

Tanglertpaibul–Rao's equation (Tanglertpaibul & Rao, 1987):

(9) $$\eta_{\text{rel}}=1+\left[\eta \right]C$$

Higiro's equations (Higiro, Herald, & Alavi, 2006):

(10) $$\eta_{\text{rel}}=e^{\left[\eta \right]C}$$

(11) $$\eta_{\text{rel}}=\frac{1}{1-\left[\eta \right]C}$$

where [*η*] is the intrinsic viscosity and *C* is the concentration. According these equations, intrinsic viscosity is the slope obtained by plotting *η*_esp_, ln *η*_rel_ or 1–(1/*η*_rel_) versus *C*, respectively.

### 2.4 Characterization of the hydrolysates

#### 2.4.1 HPLC analysis

Hydrolysates were analyzed by HPLC along with solutions of levan HS, fructose, ultrapure water (blank), and α‐D‐fructofuranose‐β‐D‐fructofuranose 2′,1:2,3′‐dianhydride (DFA III) based on the method of Jang et al. (2006). The apparatus consisted of an HPLC 1200 Infinity Series equipped with a Shodex KS‐802 column (Showa Denko, K. K.) using ultrapure water as the mobile phase and an Optilabrex 1260 GPC differential RI detector. The flow rate was 0.4 ml/min, and the column temperature was maintained at 50°C. Peak alignment was performed using a 0.3% pullulan standard solution. The injection volume was 100 µl.

#### 2.4.2 Electrospray ionization‐mass spectrometry (ESI‐MS)

Mass spectrometry was used to complement the HPLC results in the identification of the hydrolysis products obtained at the four different temperatures. Analysis was carried out using an ESI‐MS (Exactive, Thermo Fisher Scientific K.K.) in a mass range of *m/z* 150–2,000. Each solution was diluted with methanol. The following instrument settings were used: spray voltage, 2.6 kV; sheath gas pressure, 30 arbitrary units (a.u.); auxiliary nitrogen pressure, 15 a.u.; capillary temperature, 300°C; heater temperature, 250°C; capillary voltage, −27.5 V; tube lens voltage, −110 V; and skimmer voltage, −45 V.

## 3 RESULTS AND DISCUSSION

### 3.1 Characterizations of levan

**Figure 2:**
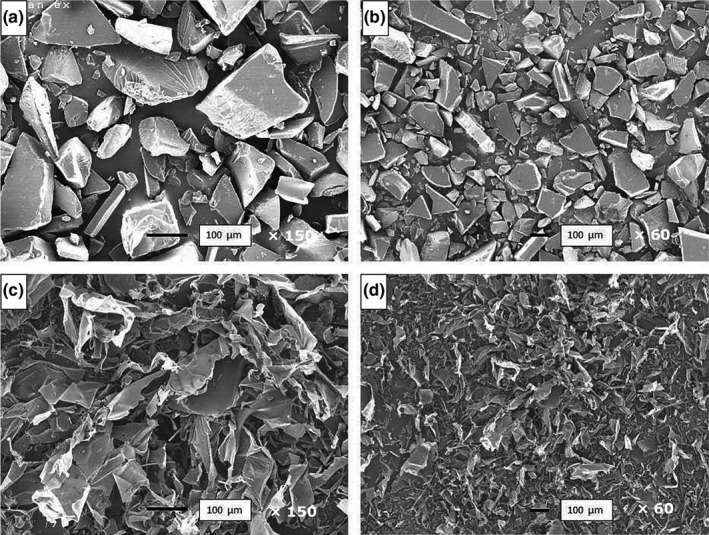
Scanning electron microscope images of levan HS at ×150 (a) and ×60 (b) magnification, and levan EH at ×150 (c) and ×60 (d) magnification

Both substrates were examined by SEM (Figure 2). Levan HS showed a crystalline morphology with relatively smooth surfaces and angular corners, as expected from its sugar‐like appearance. In contrast, levan EH showed an aggregated structure with a porous appearance and some rain drop‐shaped granules, similar to the morphology reported by Xu et al. (2018) for levan from *Brenneria* sp. EniD312 and by Kekez et al. (2016) for levan from *Bacillus licheniformis*.

Further substrate analysis was carried out using SEC‐MALS, and the molecular conformation parameters are summarized in Table 1. Integration of the peaks resulted in a molar mass on the order of 10^6^ g/mol for each levan sample. This high molar mass is in accordance with several reports on levans from bacterial sources, which contrasts to levans from plant sources (Benigar et al., 2014). For levans from bacteria, Xu et al. (2018) reported a molar mass on the order of 10^8^ g/mol for levan from *Brenneria* sp. EniD312, whereas Kazak, Ates, Ozdemir, Yalcin, and Toksoy Öner (2015) and Poli et al. (2009) reported a molar mass on the order of 10^6^ g/mol for levan polysaccharides from *H. smyrnensis*.

**Table 1:** Molecular parameters of the levan samples obtained by multi‐angle light scattering

Levan sample	*M*_w_ (g/mol)	*M*_n_ (g/mol)	Polydispersity	*R*_g_ (nm)	*R*_h_ (nm)	*R*_g_/*R*_h_	Slope of conformation plot
Levan HS	4.38 × 10^6^	3.63 × 10^6^	1.21	41.0	135.8	0.302	0.332
Levan EH	4.14 × 10^6^	3.96 × 10^6^	1.05	32.0	120.4	0.265	0.334

Abbreviations: EH, *Erwinia herbicola*; HS, *Halomonas smyrnensis*; *M*_n_, number average molar mass; *M*_w_, weight average molar mass; *R*_g_, radius of gyration; *R*_h_, hydrodynamic radius.

Levan HS presented a higher radius of gyration and hydrodynamic radius when compared with that of levan EH. Information on the molar mass, radius of gyration, and hydrodynamic radius facilitated estimation of the conformation of the samples. *R*_g_ depends on the mass distribution, whereas *R*_h_ reflects the shape of the molecules. The standard ratio between these two is ~0.778 for compact structures, for example, hard spheres, 1.78 for static coils of linear molecules and >2 for rods (Wolff et al., 2000). In the present study, the structure‐sensitive parameter, the ratio of radius of gyration and hydrodynamic radius, was approximately 0.3 for levans from both sources, although this value was not in accordance with those reported in the literature (Wolff et al., 2000). The very small value for the *R*_g_ with respect to the high molar mass values suggested that the molecules assume a compact globular conformation, as reported previously for several highly branched levans (Heyer et al., 1998). According to Runyon et al. (2014), ratios of *R*_g_/*R*_h_ smaller than 0.7 typically represent highly swollen structures or microgels.

Xu et al. (2018) reported a *R*_g_ of 30 nm based on HPSEC‐MALLS‐RI analysis of levan from *Brenneria* sp. Eni312. Those authors also reported a mean particle diameter of 176 nm for 0.1% solutions of levan analyzed by DLS (Xu et al., 2018). At higher concentrations, the measured mean particle diameter increased, which was attributed to the water solubility of levan, such that more water molecules were absorbed by intermolecular affinity, resulting in the formation of large aggregates. Using transmission electron microscopy, Xu et al. (2016) reported a spherical conformation of 20‐ to 50‐nm diameter for levan from *Paenibacillus bovis* sp., and this levan exhibited a molar mass on the order of 10^6^ g/mol.

According to Jakob et al. (2013), levans with molar masses smaller than 10^5^ g/mol exhibited a random coil conformation, whereas higher‐molecular‐mass levan molecules adopted globular conformations because of strong intramolecular interactions among more distantly located fructose residues, a phenomenon that can occur at certain molar masses. Polymer chains with critical molar masses collapse into compact coils with an overall globular shape, in which monomers have fixed positions and rotations about the bonds of the backbone are severely restricted.

The polydispersity index value in Table 1 reported reflects a narrow molar mass distribution among molecules for levan EH, similar to the result obtained for levan from *H. smyrnensis* by Kazak et al. (2015) using boric‐acid‐free medium. For levan HS, the polydispersity index value was higher than that obtained for levan EH. The levan HS value was similar to that reported by Xu et al. (2018) for levan from *Brenneria* sp. and to that reported by Szwengiel, Czarnecka, and Czarnecki (2007) using levan sucrase from *Bacillus subtilis* and *Candida cacaoi*.

**Figure 3:**
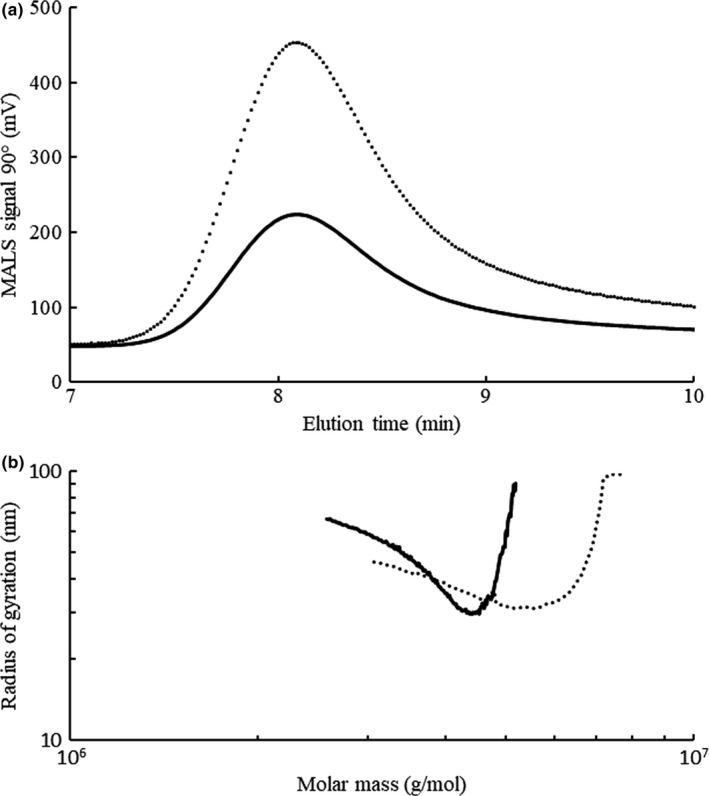
SEC‐MALS Elution profile (a) and conformation plot (b) for levan EH (∙∙∙∙∙∙) and levan HS (—)

The elution profile and the double logarithm plot of the molar mass against the *R*_g_ (conformation plot) are shown in Figure 3. The retention times were almost identical and both samples showed tails at high volumes, which suggests branching structures (Shimizu & Ushiyama, 2018). In the conformation plot, the increase in the region of lower molar masses provides a clear indication of the presence of branching, whereas from a molar mass of about 4 × 10^6^, levan HS appears to have fewer branches than levan EH because its conformation plot is shifted to higher radii (Podzimeck, 2011). This observation is in accord with the study by Kazak et al. (2015) where a very small degree of branching in levan obtained from *H. smyrnensis* was reported.

The slopes of the conformation plots were approximately 0.33. This value is consistent with the expected value for a hard sphere (Wolff et al., 2000). Using AF4‐MALS‐RI, Jakob et al. (2013) reported a slope value of 0.26 for extremely high molar mass fractions of levans from *Kozakia baliensis*. Additionally, these authors reported a slope value of 0.46 and radii of gyration of 27–34 nm for levans from *Gluconobacter frateurii* with molar masses of 4–6 × 10^6^ g/mol.

**Figure 4:**
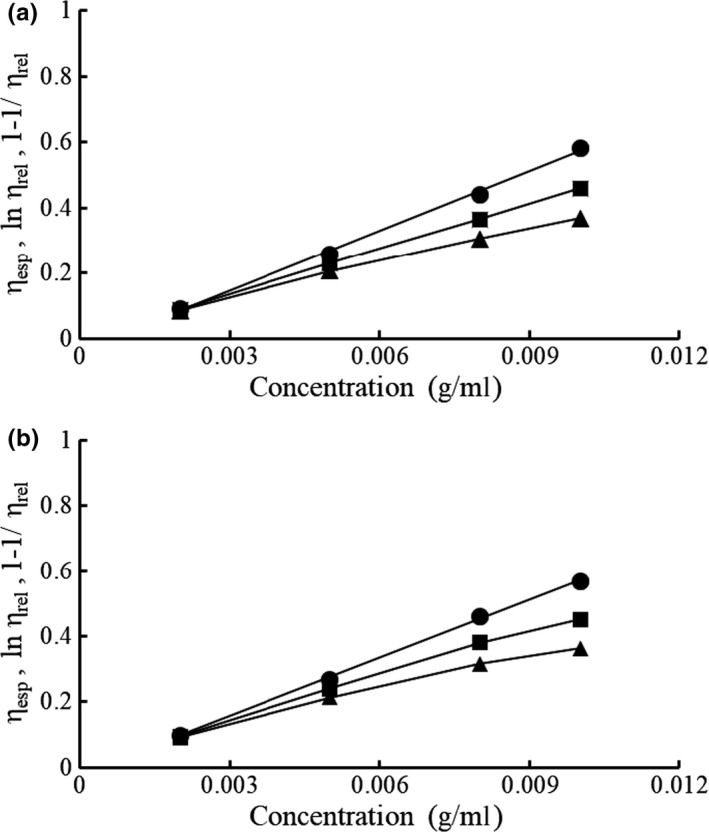
Tanglertpaibul–Rao (●), Higiro 1 (■), and Higiro 2 (▲) plots of levan HS (a) and levan EH (b) at 25°C

Intrinsic viscosity is a measure of hydrodynamic volume of macromolecules in dilute solution and can be used to gain insight into polysaccharide conformation (Liu et al., 2018). The triple Tanglertpaibul–Rao, Higiro 1, and Higiro 2 plots of levan are shown in Figure 4. The values of the intrinsic viscosity determined by Equations (7), (8), (9), (10), (11) are shown in Table 2. These values are lower than those reported for galactomannans and various gums (Feng, Yin, Nie, Wan, & Xie, 2018) but higher that the value of 14 ml/g reported for levan from *Bacillus* sp. (Arvidson, Rinehart, & Gadala‐Maria, 2006). In the case of levan HS, the values of intrinsic viscosity resulting from all three models were slightly higher than those for levan EH, consistent with the higher *R*_g_ of levan HS (Flory, 1953).

**Table 2:** Intrinsic viscosity values for the levan samples at 25°C

Levan sample	Tanglertpaibul and Rao		Higiro 1		Higiro 2	
	[*η*] (ml/g)	*R* ^2^	[*η*] (ml/g)	*R* ^2^	[*η*] (ml/g)	*R* ^2^
Levan HS	61.00	.997	46.21	.999	35.67	.997
Levan EH	59.31	.999	44.88	.997	34.28	.991

Abbreviations: EH, *Erwinia herbicola*; HS, *Halomonas smyrnensis*; [*η*], intrinsic viscosity.

### 3.2 Effect of temperature

**Figure 5:**
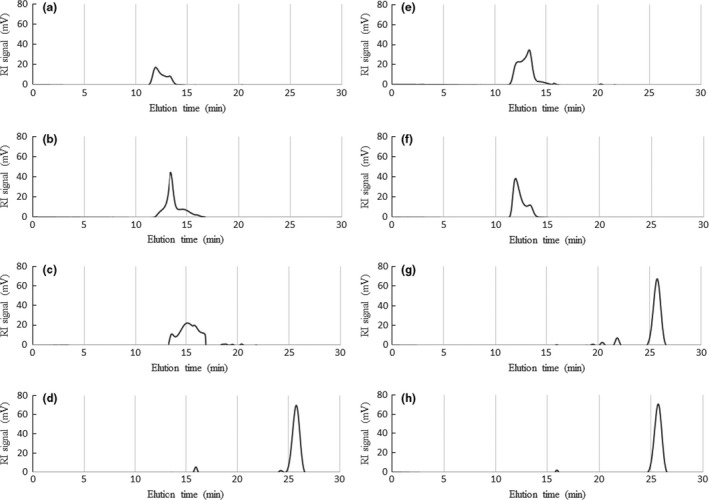
Elution profiles from gel permeation chromatography of the hydrolysis products of levan EH at (a) 120°C, (b) 140°C, (c) 160°C, and (d) 180°C, and levan HS at (e) 120°C, (f) 140°C, (g) 160°C, and (h) 180°C

**Figure 6:**
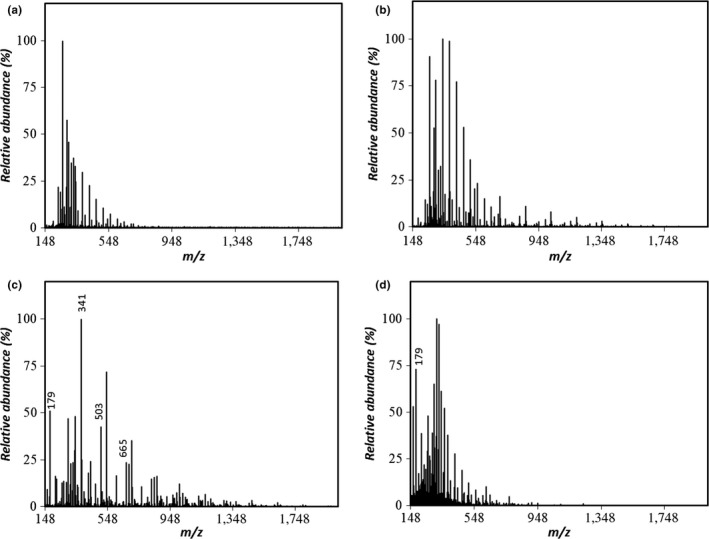
ESI‐MS spectra (negative‐ion mode) of the levan HS hydrolysate prepared by the compressed hot water process at (a) 120°C, (b) 140°C (c) 160°C, and (d) 180°C

**Figure 7:**
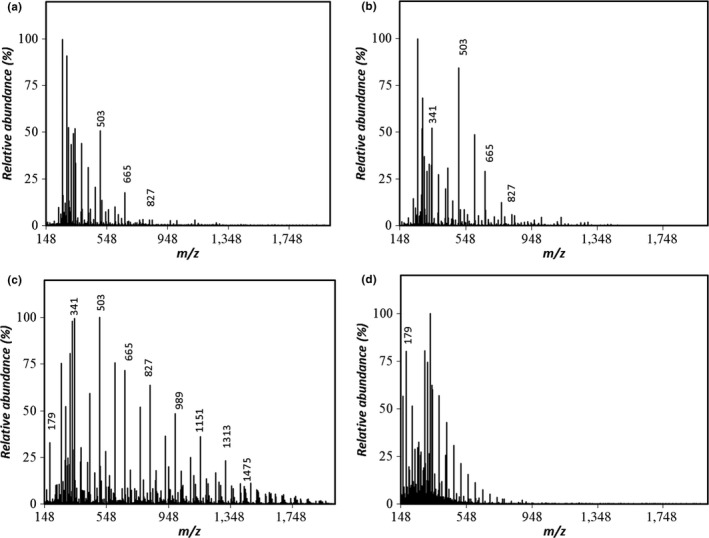
ESI‐MS spectra (negative‐ion mode) of the levan EH hydrolysates prepared by the compressed hot water process at (a) 120°C, (b) 140°C (c) 160°C, and (d) 180°C

The chromatographic mobility of both samples generated by hydrolysis at 120°C showed elution times close to the limit of the exclusion volume (Figure 5), which resembles a pattern seen for the levan standard (data not shown). This observation, combined with no recognizable ions in the range of *m/z* = 150–2,000 (Figure 6), suggests that most of levan HS was not hydrolyzed into FOS. In contrast, the 120°C hydrolysate of levan EH (Figure 7) showed peaks corresponding to GF_2_, GF_3,_ and GF_4_.

The samples generated by hydrolysis at 140°C also showed elution times close to the limit of the exclusion volume. However, the 140°C levan EH hydrolysate showed disaccharide and FOS ions with degree of polymerization (DP) values up to 5 in the ESI‐MS spectra, whereas the levan HS did not, suggesting that levan EH is hydrolyzed to FOS at lower temperatures when compared with that of levan HS.

In the chromatogram of the levan HS hydrolyzed at 160°C (Figure 5g), a monosaccharide peak eluting at the same time as the fructose standard (data not shown) dominates, with some additional fractions at shorter retention times, including a weak peak at the same time as the DFA III standard (data not shown). In contrast, the chromatogram of the levan EH hydrolyzed at 160°C (Figure 5c) showed a broad peak at shorter elution times. These differences indicate that levan HS was hydrolyzed to fructose and some oligosaccharides with lower DPs, whereas the hydrolysate of levan EH was composed of various molecular species of higher molar mass.

In the ESI‐MS spectra of levan HS at 160°C (Figure 6c), a fructose signal at *m/z* = 179 was present with other ions at *m/z* = 341, 503 and 665, corresponding to disaccharides ([F_2_‐H]^–^, [FG‐H]^–^, or [G_2_‐H]^–^) and FOS‐like 6‐kestose ([GF_2_‐H]^–^), or 6‐nystose ([GF_3_‐H]^–^) molecules.

The intensity of the fructose signal decreased for the levan EH sample hydrolyzed at 160°C (Figure 7c) with peaks corresponding to several recognizable ions. In addition to the signals at *m/z* = 341, 503, and 665, peaks were detected at *m/z* = 827, 989, 1,151, 1,313, and 1,475, corresponding to the deprotonated ions of FOSs with DPs ranging from 5 to 9. The weak peak eluting at the same time as the DFA III standard was present.

The profile of the FOSs was similar to that obtained by Itoh and Shimizu (2014) following the hydrolysis of inulin using compressed hot water fluids at 150–160°C. In the ESI‐MS spectra presented in Figures 6 and 7, both levan samples processed at 160°C yielded a signal at *m/z* = 647.21, consistent with the cluster ion of the DFA III standard. However, for both levans, this peak was weak and the amount detected in the present study was lower than that obtained by Itoh and Shimizu (2014). Even though both inulin and levan are fructans with very similar structures, under these hydrolysis conditions inulin yields DFA III, whereas levan yields mostly FOSs with a DP of 3 or higher.

For both hydrolysates generated at 180°C, only the fructose peak was left in the respective chromatogram. In both cases, this peak corresponded to a single recognizable signal at *m/z* = 179 in the ESI‐MS spectra, congruent with the presence of a monosaccharide such as fructose or glucose ([F‐H]^–^ or [G‐H]^–^).

Higher levels of branching are thought to contribute to a more rapid acid hydrolysis and a higher extent of hydrolysis in fructans. The terminal fructose units are cleaved more easily than internal ones, most likely because of a change in the conformation that the fructosyl group must adopt during hydrolysis (Barclay, Ginic‐Markovic, Cooper, & Petrovsky, 2010).

Branching leads to a contraction of the polymer chain in terms of the size and hydrodynamic volume. Thus, when comparing two samples of the same polysaccharide with similar molar masses, the root mean square radius and intrinsic viscosity of a branched sample are smaller than those of a more linear polysaccharide (Pathaweeisariyakul, Narkchamnan, Thitisak, Rungwang, & Yau, 2016), as observed when comparing levan EH to levan HS. This greater degree of branching may explain why the levan EH hydrolysate was more readily hydrolyzed.

This difference in the degree of branching may explain why the hydrolysates of levan EH were discernible at lower processing temperatures in the ESI‐MS spectra. However, at temperatures of 160°C or higher, levan HS showed higher hydrolysis to fructose, whereas the levan EH showed degradation into fractions of higher molar masses (as indicated by the chromatogram in Figure 5). Thus, in the treatment of fructans with compressed hot water fluids, the molecular conformation of the substrate must be considered when deciding the reaction conditions required to obtain the desired products.

## CONFLICT OF INTEREST

The authors declare that they have no conflict of interest.

## ETHICAL APPROVAL

This study does not involve any human or animal testing.
